# Effect of pH on growth and Cd accumulation in different rice varieties under hydroponics

**DOI:** 10.1080/15592324.2024.2399429

**Published:** 2024-09-04

**Authors:** Falian Lan, Xia Zou, Bao Guo, Xiaoyi Zhou, Dawei He, Zhenhua Zhang, Jin-Song Luo, Chunhua Dong

**Affiliations:** a College of Resources, Hunan Agricultural University, Changsha, Hunan, China; b Soil and Fertilizer Institute of Hunan Province, Changsha, Hunan, China; c Hunan New Type Fertilizer Engineering and Technological Research Center, Changsha, Hunan, China

**Keywords:** Rice, pH, Cd, subcellular components

## Abstract

Currently, applying lime to cadmium (Cd)-contaminated paddy fields to increase pH and reduce Cd availability is an effective method to control excessive Cd levels in rice grain. However, under hydroponic conditions, the impact of increased pH on Cd accumulation in different rice varieties remains unclear. This study employed three rice varieties (Yuzhenxiang, Shaoxiang 100, Xiangwanxian 12) with different Cd accumulation characteristics under different pH and long-term treatment with 1 μM CdCl_2_, to study the effect of pH on growth and Cd accumulation in different rice varieties. The result showed that as pH shifted from 5 to 8, the SPAD values, shoot dry weight, and plant height of the three rice varieties significantly decreased. The main root length, root volume, and root dry weight of Yuzhenxiang, and Shaoxiang100 significantly decreased. Conversely, the root architecture indicators of Xiangwanxian 12 did not change significantly. As for element accumulation, increasing the pH significantly increased the content of Mn in both the shoots and roots of all three varieties. Yuzhenxiang significantly reduced Cd content in both the shoots and roots of rice, while Shaoxiang100 significantly increased Cd content in both parts. Xiangwanxian 12 showed a significant increase in Cd content in the shoots but a decrease in the roots. In terms of subcellular distribution, Yuzhenxiang significantly reduced Cd concentrations in the cell wall and organelles of root cells, resulting in lower Cd concentrations in the root tissue. Conversely, Shaoxiang100 significantly increased Cd concentrations in the cell wall, organelles, and soluble fractions of root cells, leading to higher Cd concentrations in the root tissue. Xiangwanxian 12 also exhibited a decrease in Cd concentrations in the cell wall, organelles, and soluble fraction of root cells, resulting in lower Cd concentrations in the root tissue. Additionally, the expression of the OsNRAMP5 and OsHMA3 gene was significantly increased in Shaoxiang 100, while no significantly change in Yuzhenxiang and Xiangwanxian 12. These results provide important guidance on the impact of pH on Cd accumulation during the vegetative growth stage of different rice varieties.

## 1. Introduction

Cadmium (Cd) is a rare heavy metal present in the earth’s crust. However, in the past few decades, due to atmospheric deposition of industrial waste gases, irrigation of mining and metallurgical waste water, and the use of fertilizers and organic fertilizers, Cd pollution in farmland has become increasingly severe. Therefore, it is crucial to establish targeted control and remediation methods.^1–3^ In addition, Cd has strong mobility and toxicity and a long decomposition cycle, and is difficult to degrade, posing a significant threat to environmental safety and harmful to human health through the food chain.^4^ Currently, applying lime to Cd-contaminated paddy fields to increase pH and reduce Cd availability is an effective method to control excessive Cd levels in rice grain. It is still unclear whether increasing pH has different effects on Cd accumulation in different rice varieties.

Cd in the soil can inhibit the growth, development, and quality of rice. Many soil factors have been shown to affect plants uptake of Cd, with pH being the most significant soil factor.^5^ Plants mainly absorb the available forms of heavy metals in the soil, and soil pH can directly affect the distribution and content of available forms of heavy metals in the soil. The content of available Cd in acidic soil is negatively correlated with pH, and soil acidification increases the content of available Cd in the soil, enhances the uptake and transport of Cd by rice, and ultimately leads to Cd accumulation in rice grains.^6,7^ Between pH 4.0 and 7.7, the soil’s Cd adsorption capacity triples with each unit increase in pH, so applying alkaline substances to control soil pH is one of the most economically and effectively methods to change plant Cd uptake, accumulation, and plasmid field pollution.^8^ Under hydroponic conditions, the change of pH also significantly affected the absorption of iron and Cd in rice,^9^ but whether there are differences between varieties is unclear.

Plant roots are essential organs for Cd absorption, and many Cd uptake and transport proteins have been reported.^10^ Cd primarily enters root cells through transport systems responsible for absorbing essential elements.^11,12^ The absorption of Cd in rice is mainly mediated by NRAMP5,^13,14^ while the long-distance transport of Cd in rice is mainly regulated by the HMA3^15^and HMA2^16,17^ families. Understanding the mechanisms of Cd absorption and transport in rice is crucial for breeding low-Cd rice varieties to ensure human health and safety. Moreover, revealing the response mechanism of Cd to pH is also a key component of understanding the Cd accumulation mechanisms in rice. Changes in pH significantly affect the expression of some Cd uptake and translocation genes in rice. For example, at pH 6, OsNRAMP1 and OsHMA2 are significantly upregulated in ZJZ17 and XZY rice varieties, showing a positive response to Cd absorption and transport in rice.^18^ At the same time, studying different plants at the subcellular level can provide deeper insights into the mechanisms of Cd accumulation and tolerance in plants. Different subcellular components of plants have different Cd accumulation capacities. Studies have shown that the cell wall plays an important role in heavy metal deposition in plant subcellular components,^19^ significantly reducing the Cd entering the cytoplasm, thereby alleviating Cd toxicity in plants.

The results of previous studies show that Yuzhenxiang is a high Cd accumulation variety, Xiangwanxian 12 is a low Cd accumulation variety.^20^ Shaoxiang 100 is a low Cd accumulation variety with mutations in Cd main uptake gene *NRAMP*5.^21^ This study employed these rice varieties to study the effect of pH on growth and Cd accumulation in different rice varieties. Under the treatment of 1 μM Cd, the nutrient solution with pH 5 simulated the acidic farmland soil, and the nutrient solution with pH 8 simulated the soil applied with alkaline substances. Meanwhile, by investigating the accumulation of Cd in rice root subcellular components and the expression of key Cd absorption and transport genes *OsNramp5* and *OsHMA3*, this study aims to further understand the regulatory mechanism of pH on Cd accumulation in rice.

## 2. Materials and methods

### 2.1. Rice materials and treatments

Rice seeds of high Cd accumulation cultivars Yuzhenxiang, low Cd accumulation cultivars Xiangwanxian 12, and Cd absorption main effective gene OsNRAMP5 mutation Shaoxiang 100varieties provided by Hunan Academy of Agricultural Sciences were used as test materials.^20,21^ The rice seeds were washed with distilled water, then soaked in deionized water for 2 days and germinated at 37°C for 16 hours. The uniformly germinated seeds were sown in 96-hole PCR plates with the bottom cut off and floated on the surface of deionized water. After 7 days, the seedlings were transplanted into 5 liter pots contain Yoshida solution with pH values of 5 and 8, respectively (0.37 mM CaCl_2_, 0.17 mM NaH_2_PO_4_, 0.47 mM MgSO_4_, 0.27 mM K_2_SO_4_, 0.70 mM (NH_4_)_2_SO_4_, 45 mM Fe-EDTA, 0.40 mM SiO_2_, 15 mM H_3_BO_3_, 4.6 mM MnSO_4_, 0.1 mM NaMoO_4_, 0.15 mM ZnSO_4_, 0.16 mM CuSO_4_). Rice seedlings are updated with nutrient solution every four days. In order to maintain pH stability, 0.05% 2-Morpholino ethanesulfonic acid (MES) buffer was added to the culture solution and pH changes were monitored daily, and diluted hydrochloric acid and sodium hydroxide are used to adjust the pH value of the nutrient solution to 5 and 8, supplemented with CdCl_2_ to achieve a Cd concentration of 1 μM in the nutrient solution. The rice seedlings were grown in a greenhouse under a 14-hour light/10-hour dark cycle, a light intensity of 300–320 μmol/m2/s, and a humidity of 60–75%. Rice growth parameters were measured after 30 days.

### 2.2. Measurement of plants SPAD values，leaf area and biomass

SPAD values（chlorophyll content）were measured at the topmost leaf tips of each rice plant using a SPAD meter(SPAD-502PLUS) at 11 am. Ten measurements were taken at the same leaf tip of each rice plant, and the average value was recorded. Leaf area was measured using a leaf area scanner (CI-202 Portable Laser Leaf Area Meter), with the topmost leaves of each rice plant selected for measurement. The rice plants were dried at 75°C, and the weights of the roots and stems and leaves were determined using a 1-in -10,000 balance.

### 2.3. Scanning of the root system and data analysis

The rice root system was cut and rinsed with clean water, and appropriate amount of water was added to the sample tank of the root scanner (MICROTEK Scan Maker i800plus), and the cleaned root system was spread out in the sample tank to avoid overlapping of the root system as much as possible. The root system was scanned using LA-S Root Scanning Software, and the scanned images were analyzed in the software for root conformation analysis.

### 2.4. Determination of elemental concentrations

Rice plants treated with 1 μM CdCl_2_ concentration under two pH conditions were immersed in 1 μM CaCl_2_ solution and washed three times with deionized water. Each plant represented a sample, with four replicates set. After drying in a 75°C oven for 1 day, the samples were weighed and digested overnight with ultra-pure HNO_3_, followed by boiling at 100°C for 2 hours. After dilution and filtration, the Cd concentration was measured using ICP-MS (PerkinElmer Nexion350) as previously described.^22^

### 2.5. Extraction and determination of subcellular components in rice

Subcellular components were extracted using differential centrifugation. Fresh samples were weighed and ground into a slurry in extraction solution (250 mmol/L sucrose; 50 mmol/L Tris-HCl, pH 7.5; 1 mmol/L dithiothreitol). The slurry was centrifuged at 300 g for 30 seconds to obtain the cell wall residue. The supernatant was further centrifuged at 20,000 g for 45 minutes to obtain the organelle pellet, while the soluble fraction was collected from the supernatant. The extracted cell wall and organelles samples were dried, digested with ultra-pure HNO_3_, and boiled at 100°C for 2 hours. After dilution and filtration, the Cd concentration was measured using ICP-MS. The soluble fraction was directly boiled with HNO_3_, diluted, filtered, and analyzed for Cd concentration using ICP-MS.

### 2.6. RNA extraction and gene expression identification

Total RNA was extracted from rice using Trizol reagent (Invitrogen), and cDNA synthesis was performed using the HiscriptII RT SuperMix Kit (Vazyme). QRT-PCR was carried out using ChamQTM SYBR Color qPCR Master Mix (Vazyme) and Step One Plus Real-Time PCR system. The relative expression levels of genes were calculated using the 2^−ΔCt^ method. Primers used for gene expression detection are shown in Table 1.

**Table 1.** Related primer sequences.

Primer name	Forward	Reverse
Ub	GCTCCGTGGCGGTATCAT	CGGCAGTTGACAGCCCTAG
OsNRAMP5	AGCAGCAGTAAGAGCAAGATGG	GGGGAGGTCGTTGTGGATG
OsHMA3	CCATGGCATCTGTCCTGGTT	TCCGGAACTGTTTCGCATGA

### 2.7. Data processing and analysis

The experimental data were statistically analyzed using IBM SPSS Statistics 2022 with at least four biological replicates for each experiment. Tukey’s honest significant difference (HSD) tests were used for comparisons (*p* < 0.05). Different lowercase letters on the column indicate significant differences between different treatment and varieties at the *p* < 0.05 level. Bar graphs and gene expression plots were made using Origin2021 software, and three-line tables were drawn in Excel. Correlation analysis was carried out using Pearson Correlation Coefficient of SPSS software.

## 3. Results

### 3.1. Increasing pH suppresses the growth of rice shoots

**Figure 1.**
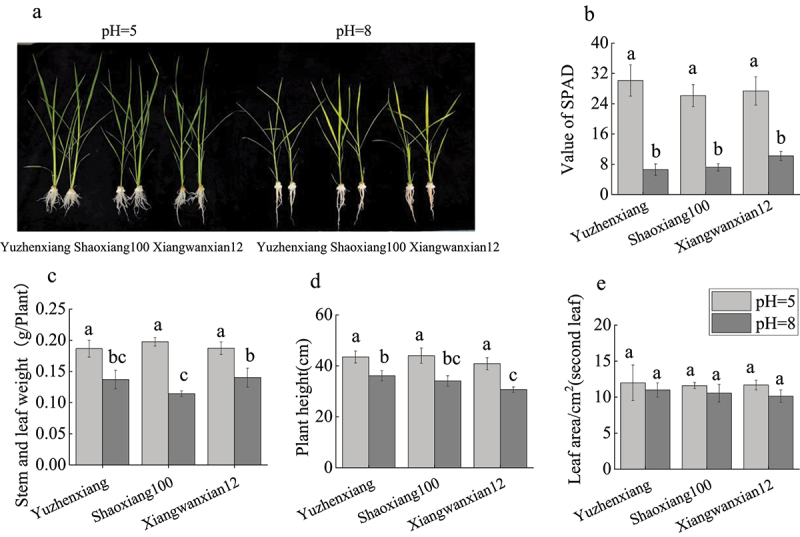
The impact of different pH levels on the shoot growth of different rice varieties under long-term low Cd concentrations. The growth phenotypes of Yuzhenxiang, Shaoxiang 100 and xiangwanxian 12 at pH = 5 and pH = 8 (a). SPAD values (b), stem and leaf weight (c), plant height (d) and leaf area (e) were measured for Yuzhenxiang, Shaoxiang 100, and xiangwanxian 12 at PH = 5 and PH = 8. The data represent the mean ± SD, *n* ≥ 3. Different letters on the column indicate significant differences between different treatment and varieties at the *p* < 0.05 level.

To investigate the effects of different pH levels on the shoot growth of different rice varieties under 1 μM CdCl_2_ concentration background, various parameters of rice seedlings grown under different pH conditions were measured. The results revealed significant differences in rice growth under different pH conditions (Figure 1a). With the transition from pH 5 to 8, the SPAD values, shoot biomass, and plant height of rice plants of all varieties significantly decreased (Figure 1b–d), while there was no significant change in leaf area for each variety (Figure 1e). Correlation analysis showed that leaf SPAD, shoot dry weight, plant height and leaf area were negatively correlated with pH increase, but not with varieties (Table 2). These results indicate that pH can significantly alter the growth status of rice plants under 1 μM CdCl_2_ stress.

**Table 2.** Correlation analysis of pH and variety with shoot growth characteristics of rice.

Correlation coefficient	Value of SPAD	Stem and leaf weight	Plant height	Leaf area
pH	-0.956**	-0.922**	-0.881**	-0.545**
Varieties	0.03	0.025	-0.25	-0.213

**means extremely significant correlation between pH with shoot growth characteristics at *p* < 0.01 level.

### 3.2. Increasing pH alters rice root architecture

**Figure 2.**
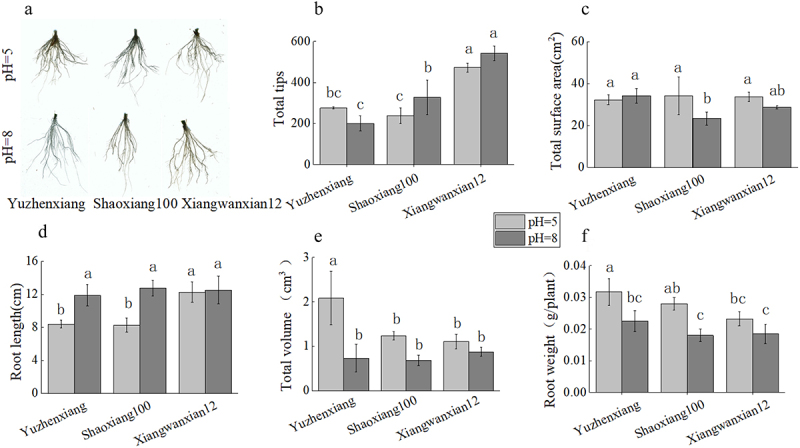
The effect of different pH levels on the root growth of different rice varieties under long-term low to moderate Cd concentrations. Root scans of Yuzhenxiang, Shaoxiang 100 and xiangwanxian 12 were conducted for pH = 5 and pH = 8 (a) At pH = 5 and pH = 8, total tips (b) total surface area (c) root length (d) total volume (e) and root weight (f) was measured. The data represent the mean ± SD, *n* ≥ 3. Different letters on the column indicate significant differences between different treatment and varieties at the *p* < 0.05 level.

With the change in pH from 5 to 8, significant change in root architecture were observed in the high Cd accumulation rice variety Yuzhenxiang and the Cd uptake gene mutation variety Shaoxiang 100 (Figure 2a). Specifically, there were no significant changes in the number of root tips and root surface area for both rice varieties (Figure 2b,c), but the primary root length significantly increased (Figure 2d), while root volume and root dry weight significantly decreased (Figure 2e,f). In contrast, for the low Cd absorption variety Xiangwanxian 12, there were no significant changes in root-related parameters with the change of pH. Correlation analysis showed that root surface area, root length, root total volume and root dry weight were negatively correlated with pH increase, the number of root tips and root length were positively correlated with variety, and root dry weight was negatively correlated with variety (Table 3). These findings suggest that under 1 μM CdCl_2_ stress, the root architecture of Yuzhenxiang and Shaoxiang 100 varieties was more sensitive to the change of pH value, but Xiangwanxian 12 was not sensitive.

**Table 3.** Correlation analysis of pH and varieties with rice root architecture.

Correlation coefficient	Total tips	Total surface area	Root length	Total volume	Root weight
pH	0.325	−0.462*	0.635**	−0.632**	−0.729**
Varieties	0.551**	−0.169	0.424*	−0.302	-0.456*

*means significant correlation between pH or varieties with rice root architecture at *p* < 0.05 level, **means extremely significant at *p* < 0.01 level.

### 3.3. Increasing pH has differential effects on the accumulation of Cd and other metal elements in different rice varieties

**Figure 3.**
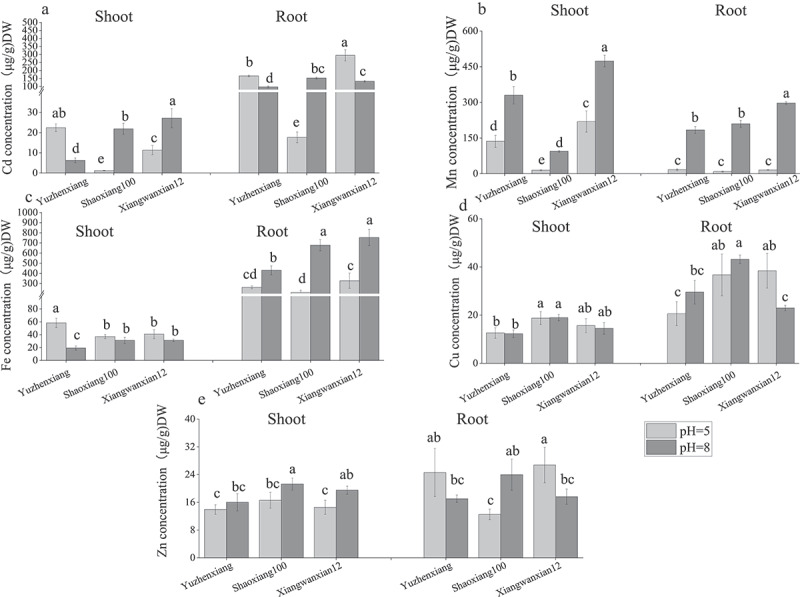
The impact of different pH levels on the accumulation of Cd and other metallic elements in different rice varieties. Cd concentration (a) Mn concentration (b) Fe concentration (c) Cu concentration (d) and Zn concentrations (e) were measured in the dry weight of roots and shoots of Yuzhenxiang, Shaoxiang 100 and xiangwanxian 12 at pH = 5 and pH = 8. Cd contents were determined by using an inductively coupled plasma mass spectrometry (ICP-MS). The data represent the mean ± SD, *n* ≥ 3. Different letters on the column indicate significant differences between different treatment and varieties at the *p* < 0.05 level.

To investigate the effects of different pH levels on the accumulation of Cd and other metal elements in various rice varieties, samples were taken after the rice plants reached the tillering stage to measure the concentrations of Cd and other metal elements in different parts of the plants under two pH conditions. The study found that, under pH 5 conditions, the Cd concentration in the shoots of the Yuzhenxiang variety was higher than that of Shaoxiang 100 and Xiangwanxian 12 (Figure 3a), which is consistent with previous study result.^20,21^ With the change in pH from 5 to 8, the Cd concentration in both the shoots and roots of the high Cd-absorbing variety Yuzhenxiang decreased significantly (Figure 3a). In contrast, the Cd concentration in both the shoots and roots of the Shaoxiang 100 variety, which lacks the major gene for Cd absorption, increased significantly, while the Cd concentration in the shoots of Xiangwanxian 12 increased significantly but decreased significantly in the roots (Figure 3a). Increasing the pH significantly increased the content of Mn in both the shoots and roots of all three varieties (Figure 3b) and significantly increased the content of Fe in the roots of all three varieties (Figure 3c). However, it significantly decreased the content of Cu and Zn in the roots of Xiangwanxian 12 (Figures 3d,e). Correlation analysis showed that the contents of Mn, Fe and Zn were significantly correlated with the increase of pH, while the contents of Cd and Cu were not significantly correlated with the increase of pH, and the contents of these elements were not significantly correlated with the varieties (Table 4). These results indicate that increasing the pH has significant differences effects on the accumulation of elements in different rice varieties.

**Table 4.** Correlation analysis of pH and variety factors with metal content in shoot and root of rice.

Correlation coefficient	Cd in shoot	Cd in root	Mn in shoot	Mn in root	Fe in shoot	Fe in root	Cu in shoot	Cu in root	Zn in shoot	Zn in root
pH	0.354	−0.193	0.560**	0.957**	−0.725**	0.862**	−0.065	−0.001	0.710**	−0.147
Varieties	0.208	0.400	0.304	0.177	−0.088	0.366	0.327	0.250	0.239	0.097

**means extremely significant correlation between pH with metal content in shoot and root of rice at *p* < 0.01 level.

### 3.4. Increasing pH has differential effects on Cd content in subcellular components of rice roots among different varieties

**Figure 4.**
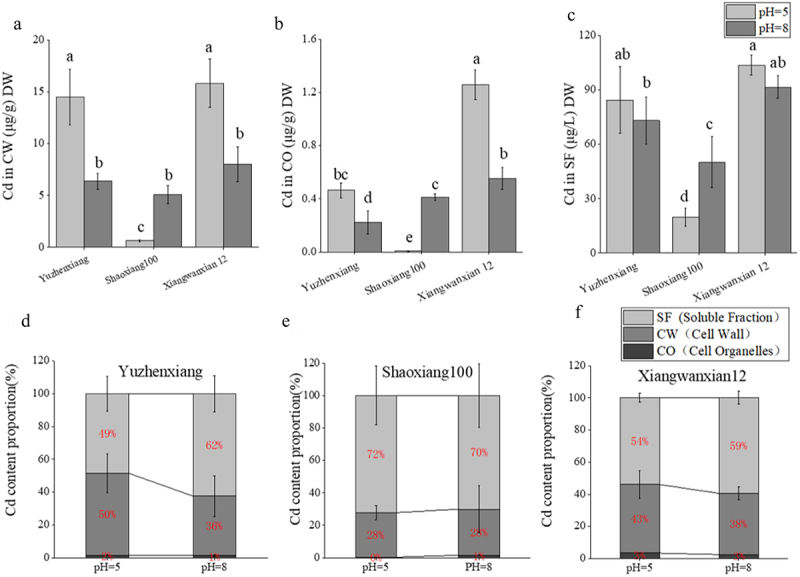
Effect of different pH on the accumulation of Cd in subcellular fractions of the root system of different rice varieties. Cadmium concentrations in cell wall (a) organelles (b) and soluble fraction (c) was measured at pH = 5 and pH = 8 for Yuzhenxiang, shaoxiang 100 and xiangwanxian 12 on dry weight. Under long-term Cd stress, the Cd content and percentage of different subcellular components of root tissues of different rice varieties were determined. The percentage of Cd content in the cell wall, organelles, and soluble fraction of the three rice varieties YuzhenXiang (d) shaoxiang 100 (e) and xiangwanxian 12 (f) was measured at pH = 5 and pH = 8. Cd contents were determined by using an inductively coupled plasma mass spectrometry (ICP-MS). The data represent the mean ± SD, *n* ≥ 3. Different letters on the column indicate significant differences between different treatment and varieties at the *p* < 0.05 level.

For the high Cd absorption variety Yuzhenxiang, there was a significant decrease in Cd content in the cell wall and organelles with the transition from pH 5 to 8, while there was no significant effect on the soluble fraction of the cell (Figure 4a–c). For the low Cd absorption variety Xiangwanxian 12, there was a significant decrease in Cd concentration in the cell wall, organelles, and soluble fraction of the cell with the change in pH from 5 to 8 (Figure 4a–c). In the case of the Cd absorption main effective gene mutation variety Shaoxiang 100, there was a significant increase in Cd concentration in the cell wall, organelles, and soluble fraction of the cell as pH changed from 5 to 8 (Figure 4a–c). Under long-term Cd stress, the Cd content and its proportion in different subcellular fractions of root tissues of different rice varieties were determined. It was observed that more Cd was distributed in the soluble fraction and cell wall, with the least proportion found in organelles (Figure 4d–f). The accumulation of Cd in different subcellular fractions of the root system of the three rice varieties was significantly affected by different pH levels, further confirming the different response of Cd accumulation in rice root tissues to pH at the subcellular level.

### 3.5. Increasing pH significantly upregulates the expression of Cd uptake and vacuolar sequestration genes in the roots of Shaoxiang 100

**Figure 5.**
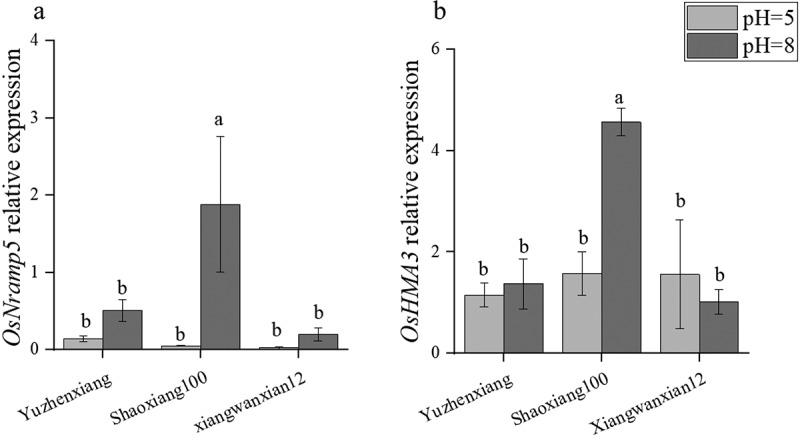
Effects of different pH on the expression of Cd uptake and transport-related protein genes in different rice varieties. At PH = 5 and PH = 8, the expression levels of OsNramp5 and OsHMA3 in the Yuzhenxiang, Shaoxiang 100 and xiangwanxian 12. The data represent the mean ± SD, *n* ≥ 3. Different letters on the column indicate significant differences between different treatment and varieties at the *p* < 0.05 level.

To investigate the effect of different pH levels on the expression of key genes related to Cd uptake and transport in different rice varieties under 1 μM Cd concentration background, real-time fluorescence quantitative PCR experiments were conducted on the relevant materials. The study found that the expression of the gene *OsNRAMP5*, responsible for Cd and Mn uptake, increased in three rice varieties as pH changed from 5 to 8 (Figure 5a). The gene *OsHMA3*, involved in Cd vacuolar sequestration in Shaoxiang 100, showed a significant increase in expression as pH changed from 5 to 8, but had no significant effect on the expression level in Yuzhenxiang and Xiangwanxian 12 (Figure 5b). With the increase of pH, the up-regulated expression of *NRAMP5* may be the reason for the significant increase of Mn content in the three rice varieties, and the molecular mechanism of the change of Cd and other metal elements still needs to be further studied in rice.

## 4. Discussion

Cd is a highly toxic heavy metal that affects plant cell division and metabolism, seriously jeopardizing plant growth, development, and physiological functions.^23^ Cd accumulation also significantly affects the shoot growth of rice plants. In this study, as pH changed from 5 to 8 under mild Cd concentration background, the shoot growth of the three rice varieties was significantly affected consistently: specifically, the plant height, biomass, and SPAD values of rice plants significantly decreased, indicating significant inhibition of their growth (Figure 1). Plant roots are important organs for Cd absorption, and thus, rice roots undergo changes in root morphology to alleviate Cd absorption and mitigate Cd toxicity under Cd stress. In this study, as pH changed from 5 to 8 under mild Cd concentration background, the main root length, root volume, and root dry weight of Yuzhenxiang and Shaoxiang 100 significantly decreased, while there was no significant effect on root tip number and total root surface area (Figure 2). The root system of Xiangwanxian 12 did not undergo significant changes. This is consistent with the results previously reported that high pH inhibits rice growth under hydroponic conditions.^18^

After rice absorbs Cd from the roots, a small portion of Cd is transported to the shoot parts and accumulates in leaves and stems. However, different rice varieties exhibit differences in Cd uptake and accumulation, leading to varying degrees of tolerance to Cd stress. Many soil factors have been shown to influence plant Cd uptake. Soil pH is usually the most significant influencing factor. In general, as soil pH decreases, the bioavailability and mobility of Cd increase because lower pH promotes the transformation of Cd from fixed forms to more biologically available forms (such as exchangeable forms).^24^ Overall, all soil processes controlling Cd behavior are particularly important in the rhizosphere. Under hydroponic conditions, the accumulation of cadmium in rice can be reduced by extreme acid or alkali environment, the Cd accumulation was the highest from a near-neutral (pH 6.0) medium, driven by the upregulation of the genes *OsNRAMP1* and *OsHMA2*.^18^ The latest study showed that soil pH is a major factor influencing mineral uptake in rice straw and grains, and genetic factors, flowering stage factors, and their interaction with soil pH contribute in a combined manner.^25^ As pH changed from 5 to 8 Yuzhenxiang significantly reduced Cd content in both the shoots and roots of rice, while Shaoxiang100 significantly increased Cd content in both parts (Figure 3A). Xiangwanxian 12 showed a significant increase in Cd content in the shoots but a decrease in the roots (Figure 3A). These results indicate that the effect of pH on Cd accumulation is a complex trait and different among rice varieties, the specific mechanism of this need to be further studied by genetic method.

When Cd enters rice cells, the cell wall is the first barrier to protect rice from Cd toxicity, and its fibers, hemifibers and pectin can prevent Cd from entering the protoplasmic layer,^26^ when a small amount of Cd enters the protoplasmic layer of plant cells, only a small portion of it will be in the form of the free state in the cells, and most of it will be chelated with a number of small molecular compounds to the form of the soluble portion of the soluble fraction.^27^ The accumulation of Cd in the subcellular fractions of different plants is not the same: for the subcellular fractions of the Cd content of winter wheat, the soluble fractions > the cell wall fractions > the cellular components;^28^ For the Cd content of subcellular fractions in rice, cell wall > soluble fraction > organelles,^29^ but some studies also showed that the distribution of Cd content was soluble fraction > cell wall >organelles,^30^ which may vary with rice varieties. Further analysis of the Cd concentrations in the subcellular fractions of the three varieties of rice revealed that Yuzhenxiang significantly reduced the Cd concentrations in the cell wall and organelles of the root cells, Xiangwanxian 12 significantly decreased the Cd concentration in the cell wall and organelles of root cells and soluble fraction and decreased the Cd concentration in root tissues, while Shaoxiang 100 significantly increased the Cd concentration in the cell wall and organelles of root cells and soluble fraction and increased the Cd concentration in root tissues (Figure 4). Although the Cd content of root cell subcellular fractions of three rice varieties was significantly altered under the influence of different pH, the Cd proportion of root cell subcellular fractions was soluble fraction > cell wall > organelles in all of them (Figure 4). This was consistent with the Cd accumulation data in root tissues of three rice varieties.The subcellular distribution of Cd was significantly different in the roots of the three rice varieties, with more than 95% stored in soluble parts and cell walls.

Cd absorption and accumulation in rice are regulated by multiple processes, including Cd absorption by roots from the soil, Cd transport from the root to the shoot parts, retransport from the xylem to the phloem, redistribution in shoot organs, and Cd accumulation in grains.^31–33^ Many root Cd absorption proteins have been reported, such as the OsNRAMP5 protein, which is polarly localized at the distal ends of the root epidermis and endodermis and has affinity for Cd, Fe, and Mn, participating in the uptake process of these heavy metal elements.^14^ Once Cd enters plant cells, part of the Cd absorbed by the roots is excreted from the cells to the outside, such as the OsHMA9 protein acting as an “efflux pump” for heavy metals on the plasma membrane.^34^ Excess Cd can also be sequestered in the vacuole by the OsHMA3 protein, thereby reducing Cd transport efficiency from roots to shoot parts.^35^ In this study, as pH changed from 5 to 8, the expression of *OsNRAMP5* increased in Yuzhenxiang, Xiangwanxian 12 and Shaoxiang 100 varieties (Figure 5),These changes in the expression of OsNRAMP5 may be the main reason for changes in Mn concentration in rice under different pH conditions (Figure 3). However, regarding the OsHMA3 gene involved in rice Cd sequestration, the expression significantly increased in Shaoxiang 100 while there was no significant change in Xiangwanxian 12 and Yuzhenxiang (Figure 5). These changes in the expression of Cd main uptake and vacuolar sequestration transport genes may be the partly reason for changes in Cd concentration in rice under different pH conditions. To reduce Cd accumulation in grains, many solutions have been proposed, such as raising soil pH, optimizing irrigation measures, and developing low Cd accumulation rice varieties.^36^ Based on our experimental results, there are differences in the effects of increased pH on Cd accumulation among different rice varieties, and the specific mechanisms remain to be further elucidated.

## 5. Conclusions

This study investigated the effects of pH on rice growth, Cd content in shoot and root, subcellular distribution of Cd in root tissues, and differential expression of Cd absorption and transportation genes. The results showed that when pH of hydroponic nutrient solution ranged from 5 to 8, the growth of shoot and root of rice was significantly inhibited, leaf yellowing and root configuration changed. Mn content was significantly increased in both shoot and root of rice, and the expression of *NRAMP5*, a gene responsible for Mn uptake was up-regulated. The absorption and translocation process of Cd, and the distribution of Cd in root different subcellular components were significantly different among three rice varieties. These results indicate that there are significant differences in the effects of pH on Cd accumulation in different rice varieties, providing a reference for strategies to reduce Cd in rice. Why does the response of Cd accumulation in rice to pH vary among varieties? Study the specific molecular regulatory mechanism is a very interesting research in future.

## Data Availability

Enquiries about data availability should be directed to the correspondence authors.
